# Sleep Disorders in Patients With Craniopharyngioma: A Physiopathological and Practical Update

**DOI:** 10.3389/fneur.2021.817257

**Published:** 2022-02-09

**Authors:** Andrea Romigi, Tiziana Feola, Simone Cappellano, Michelangelo De Angelis, Giacomo Pio, Marco Caccamo, Federica Testa, Giuseppe Vitrani, Diego Centonze, Claudio Colonnese, Vincenzo Esposito, Marie-Lise Jaffrain-Rea

**Affiliations:** ^1^Neuromed Institute, Istituto di Ricovero e Cura a Carattere Scientifico, Pozzilli, Italy; ^2^Department of Experimental Medicine, Sapienza University of Rome, Rome, Italy; ^3^Human Neurosciences, Sapienza University of Rome, Rome, Italy; ^4^Department of Biotechnological and Applied Clinical Sciences, University of L'Aquila, L'Aquila, Italy

**Keywords:** craniopharyngioma, sleep disorder, hypothalamic syndrome, hypothalamic obesity, obstructive sleep apnea, hypersomnia, narcolepsy, circadian rythm disorders

## Abstract

Sleep disorders (SDs) represent an important issue in patients with craniopharyngioma (CP). Nearly 70% of these patients complain of sleep-wake cycle alterations and/or excessive diurnal somnolence due to sleep-related breathing disorders, such as obstructive sleep apnea (OSA) and/or central hypersomnia, including secondary narcolepsy. SDs may severely reduce quality of life, increase disease-related cardiorespiratory and cardiovascular morbidity, and finally play a major role in increased long-term mortality reported on patients with CP. A major risk factor for SDs is represented by the hypothalamic syndrome, which may develop because of direct hypothalamic damage by the tumor itself and/or complications of the treatments, neurosurgery and/or radiotherapy, and typically includes permanent neuroendocrine dysfunctions, morbid obesity, and secondary metabolic disorders. Despite increasing attention to SDs in the general population, and in particular to OSA as a risk factor for cardio-metabolic diseases and excessive daytime somnolence, sleep evaluation is still not routinely proposed to patients with CP. Hence, SDs are often underdiagnosed and undertreated. The aim of this paper is to update current knowledge of the pathogenesis and prevalence of SDs in patients with CP and propose practical algorithms for their evaluation and management in clinical practice. Particular attention is paid to screening and diagnostic tools for appropriate characterization of SDs, identification of risk factors, and potential role of hypothalamic sparing surgery in the prevention of morbid obesity and SDs. Available tools in sleep medicine, including lifestyle interventions, drugs, and respiratory devices, are discussed, as well as the importance of optimal hormone replacement and metabolic interventions. Current limits in the diagnosis and treatment of SDs in patients with CP and possible future avenues for research agenda are also considered.

## Introduction

Craniopharyngiomas (CPs) are rare benign parasellar tumors derived from Rathke's pouch rests, and are classified into two subtypes (1). Most are adamantinomatous, typically presenting as mixed solid/cystic tumors with frequent calcifications, driven by somatic beta-catenin mutations, whereas papillary CPs are suprasellar, mostly solid, tumors with frequent BRAF mutations (2). CPs have a bimodal age distribution, with peaks of incidence occurring in pediatric (5–14 years, adamantinomatous) and adult patients (50–74 years, both) (1, 2). Despite benign histology and high overall survival (>90% in childhood-onset CP), the standardized mortality rate in patients with CP has been variably estimated from 2.88 to 9.28, with a 3- to 19-fold increase in cardiovascular mortality compared to the general population (3). Prognosis may vary according to tumor characteristics and treatment, secondary co-morbidities, and childhood vs. adult onset of the disease (1). Neuroendocrine dysfunctions include partial or complete hypopituitarism, hyperprolactinemia, and diabetes insipidus (DI). The most dramatic complication is the development of a hypothalamic syndrome (HS), which is typically associated with neuroendocrine disorders and includes neurocognitive changes (4) morbid hypothalamic obesity (HO) and related systemic complications (5), a variety of sleep disorders (SDs) including sleep-related breathing disorders (SBDs), central hypersomnia and abnormal wake-sleep circadian rhythms (6, 7), and less commonly abnormalities of thirst and central temperature or cardiovascular regulation. Hypothalamic damage may severely impair the quality of life (QoL) of patients and has an impact on long-term mortality (8). The extension and localization of hypothalamic injury due to the tumor itself, neurosurgery, and, in some cases, radiotherapy, contribute to the timing and severity of HS (5, 9). The optimal treatment for CP and related complications remains difficult and relies on a multidisciplinary approach, with increasing attention being paid in the last decades to the prevention of hypothalamic damage during surgery (2, 10).

Overall, SDs have received more attention in pediatric than in adult patients with CP, who include long-term survivors of childhood-onset CP and patients with an adult-onset disease. In pediatric cohorts, SDs have been heterogeneously reported as daytime sleepiness/hypersomnia, sleep disturbances such as difficulty to fall asleep or waking up during the night, and variably evaluated by self-assessment—specific questionnaires (11), items as a part of QoL assessment (12)—and more recently by means of sleep medicine tools aiming to better define entities such as SBDs, like obstructive sleep apnea (OSA) (7) or secondary narcolepsy (13). Within the methodological limits and heterogeneity of reported studies, the prevalence of SDs and/or excessive daytime sleepiness (EDS) approaches 70–80% (11, 13), with an adult prevalence of OSA around 40% (6). A common observation is the higher prevalence of SDs in the presence of hypothalamic involvement, with a bi-directional interplay between obesity and SDs. Indeed, SDs recognize a multifactorial pathogenesis: (1) a strict relationship with HO; (2) damage to the hypothalamic nuclei regulating sleep, wakefulness and circadian rhythm; (3) dysfunction of the pharyngeal/respiratory muscles; (4) suboptimal endocrine treatment; (5) fatigue, and psychosocial disorders (14). On the other hand, SDs contribute to and aggravate metabolic and cardiovascular co-morbidities. In fact, SDs increase the risk of insulin-resistance, obesity, and diabetes mellitus (DM) (15), and intermittent hypoxemia in OSA is an independent risk factor for cardiovascular and cardiorespiratory mortality (16). SDs may also represent a risk factor for neurocognitive decline (17) and cancer (18).

Nonetheless, SDs remain largely underdiagnosed in clinical practice, which may be explained by the complex clinical management of patients with CP and insufficient awareness or access to centers for sleep medicine. However, no guidelines are available for the diagnosis and management of SDs in such patients. Because increasing attention is currently being given to sleep health in the general population because of relevant health and socioeconomic consequences (e.g., reduced performance at work, driving safety, and social relationship), we wished to review the current knowledge of SDs in patients with CP and propose, based on our multidisciplinary experience, practical algorithms for the screening, diagnosis, and management of such conditions. Current limits and future therapeutic options will also be discussed.

## Classification and Pathogenesis of Sleep Disorders in Patients With CP

Sleep disorders (SDs) are classified according to the third edition of the International Classification of Sleep Disorders (ICSD-3) of the American Academy of Sleep Medicine (19), which identifies seven major categories of disorders: insomnia disorders, SBDs, central disorders of hypersomnolence, circadian rhythm sleep-wake disorders (CRSWDs), sleep-related movement disorders, parasomnias, and other SDs. The most frequently reported SDs in CP are EDS (11, 20), central hypersomnia and secondary narcolepsy (13, 14, 21–27), SBDs (7, 24), and CRSWD (28–30), which require appropriate characterization and understanding of their underlying causes. Since clinical pictures may be complex and different elements may co-exist in the same patient, this may be achieved through a specific expertise in sleep diseases. Although the real prevalence of SDs before and after CP surgery/radiotherapy should be clarified, Mandrell et al. (13) showed that 45% of a large sample of pediatric patients with CP were affected by hypersomnia due to a medical disorder and 35% by narcolepsy, and that the main predictor of sleepiness was obesity. In addition, 80% of this cohort complained of EDS at diagnosis or after neurosurgery. This is in keeping with previous studies reporting that children and adolescents with CP complain of somnolence, fatigue, and sleep–wake disruption, which persist in 65–80% of the cases after treatment (20, 31). Similar data were reported in unselected adults with CP, with a prevalence of SDs around 70% (10). Of note, EDS may reduce work/education performance in 43% of patients with CP (6).

From a physiopathological point of view, sleep can be altered because of tumor growth toward structures involved in the control of sleep and wake, direct or indirect/vascular treatment-related injury to the same structures, or as a consequence of HO. Therefore, hypothalamic dysfunction plays an essential role in the development of SDs. Damage to the suprachiasmatic nucleus (SCN), the central biological “master clock,” leads to abnormal circadian rhythms and sleep-wake cycles. The SCN is on the neural way of control of the nocturnal pineal secretion of melatonin (32). It is composed of 20.000 neurons and glia that change their rate of firing in response to variation in light (33) and modulates several processes, such as sleep and food intake. Internal rhythm is strictly linked to external light cycle, and CRSWD develop when misalignment between light cycle and internal rhythm occurs. Circulating melatonin is mostly of central origin, and abnormally low nocturnal melatonin levels have been reported in patients with childhood-onset CP in association with daytime somnolence (34) and disrupted circadian rhythm (29). Similar findings were reported in a population of predominant adult-onset CP in association with reduced sleep time and efficiency, and a tendency for increased diurnal sleepiness and impaired physical health (30). Of note, chronobiotic effects of melatonin go far above sleep induction and include several systemic effects, leading to endocrine/metabolic dysfunctions in the presence of melatonin deficiency (32). Most of the master clock genes involved in circadian rhythmicity and circadian rhythm integrity are also tightly linked to metabolism and weight control (35, 36). Damage to the lateral hypothalamus, ventrolateral preoptic area, and median preoptic nucleus may impair the secretion of hypocretins, also called orexins, which are deficient in narcolepsy type 1 (37). Orexin/hypocretin-secreting neurons have broad projections to the brain and play an essential role in the promotion of wakefulness; their loss induce secondary REM sleep dysregulation, with excessive diurnal somnolence and sleep attacks by abrupt transitions from NREM to REM sleep leading to narcolepsy (38). Additional manifestations of narcolepsy (i.e., cataplexy, hypnagogic hallucinations, and sleep paralysis), an expression of fast intrusion of REM sleep or REM atonia, are less frequent in patients affected by secondary narcolepsy due to suprasellar tumors (39). Almost 38%, 15%, and 7% of patients with CP may develop cataplexy, sleep paralysis, and hallucinations, respectively (39).

### Sleep-Related Breathing Disorders

Sleep-related breathing disorders (SBDs) are the most common SDs among children and adolescents (40, 41), with 1–4% of unselected children suffering from OSA, and rising up to 13–60% in obese children (42). SBDs are also highly prevalent in adults. OSA can be recognized by polysomnography (PSG) or home sleep apnea test (HSAT), based on the Apnea/Hypopnea Index (AHI, expressed in events/h). According to the Wisconsin Sleep Cohort, ~13% of men and 6% of women have moderate-to-severe sleep apnea (AHI>15/h), and 14% of men and 5% of women have AHI ≥ 5/h plus symptoms of daytime sleepiness, both increasing with age and body mass index (BMI) (43). As a consequence, these estimates have grown substantially over the last two decades, largely because of the rising obesity epidemic (43). Adult and pediatric patients show different presentation, diagnostic criteria, course, and complications. According to the ICDS-3 (19), pediatric OSA is characterized by intermittent complete or partial obstruction (obstructive apnea or hypopnea); prolonged partial upper airway obstruction; or both prolonged and intermittent obstructions that disrupt normal ventilation during sleep, normal sleep patterns, or both (at least one obstructive, mixed apnea, or hypopnea per hour of sleep). The presence of SBD symptoms in combination with an AHI of ≥ 1/h has been applied to define pediatric OSA in most published studies (44). In adults, the definition is based on AHI ≥ 5/h, and OSA is characterized by predominant obstructive respiratory events (obstructive and mixed apneas, hypopneas, or respiratory effort related arousals). Of note, no specific criteria are considered for the definition of OSA in the “transitional” age from childhood to adulthood, which represents a significant proportion of adolescent/young adult patients with CP. The clinical presentation of OSA may differ between adult and pediatric patients. In adults, snoring and breathing irregularity in sleep may easily suggest the presence of OSA, whereas in children EDS is less perceived and may manifest as irritability, impulsivity, and distractibility (45). EDS manifests only in minority of children with OSA (46, 47). OSA is also more common in children with neurological impairment due to hypotonia of pharyngeal muscle or inability to change position during sleep (48).

SDs, in particular SBDs, may also be linked to HO, which in turn could be worsened by disrupted sleep patterns. Damage to the ventromedial hypothalamus and arcuate nucleus, which regulate hunger, satiety, and energy balance, is considered as the main determinant of HO (5). Increased energy intake and hyperphagia are not sufficient to explain HO, which is also due to imbalance between increased parasympathetic activity promoting hyperinsulinemia, reduced sympathetic activity leading to a reduced energy expenditure, and reduced daily activities because of somnolence, neurological sequelae such as visual loss, and psychological distress (5). Somnolence itself contributes to lower energy expenditure and increases appetite, leading to weight increase (49). Rapid and severe weight gain, which is typically maximal during the first 12 months following surgery, aggravates psychological distress, inactivity, and deleterious food intake, sustaining a dramatic vicious cycle in patients with CP, although BMI tends to stabilize later on. OSA has been reported in 5–46% of patients with CP (6, 7, 11, 13, 14, 23, 29, 50), depending on demographic characteristics (Table 1). Obesity is a well-known risk factor for OSA in the general population, and no significant difference was found in the prevalence of OSA between adults with CP (46%) and matched overweight and obese controls (61%) (6). However, BMI did not correlate with either the AHI or Epworth Sleepiness Scale (ESS), and diurnal somnolence was higher in adult patients with CP than in obese controls (71.5 vs. 17%), confirming that OSA is only one of several causes of somnolence in these patients, and that obesity alone does not explain the prevalence of OSA (6). Compared with adolescent obese controls, obese adolescents with CP fall asleep quicker (lower sleep onset latency), tend to sleep longer (trend toward higher total sleep time), and show more severe oxygen desaturation and present more severe AHI and central apnea index (7). Since unspecified diagnostic criteria or adult criteria were applied in many pediatric patients with CP, the pediatric prevalence of OSA may be underestimated (11, 23, 29, 50).

**Table 1:** Sleep-related breathing disorders in patients with craniopharyngioma.

**References**	**Patients (*n*)**	**Study design**	**Prevalence**	**Age**	**Diagnostic criteria**	**Diagnostic tool**
Snow et al. (23)	5	C P	2/5 (40%)	11–19 yrs	Not reported	PSG
Lipton et al. (29)	3	C selected hypersomnolent patients	3/3 (100%)	15–22 yrs	Not reported	PSG
O'Gorman et al. (7)	15	CS C (obese CP vs. obese controls)	7/13 normal-mild (53.8%) 2/13 moderate (15.3%) 4/13 severe (30.6%)	10–21 yrs	Mild OSA AHI 1.5–5/h Moderate OSA AHI 5–10/h Severe OSA AHI >10/h Abnormal CAI >1/h	PSG
Crowley et al. (6)	28	C P (obese CP vs. obese controls)	11/28 (39.2%)	16–67 yrs	AHI ≥ 5/h	PSG
Manley et al. (11)	28	R U	3/7 (42%) (2/3 OSA and CSA)	Pediatric and Adult	Not reported	PSG
Mandrell et al. (13)	110	CS CO U	5/98 (5.1%)	Pediatric and Adult	AHI ≥ 2/h for pediatric patients AHI ≥ 5/h for adult patients	PSG
Niel et al. (50)	50	U P	2/10 (20%)	3–20 yrs	AHI ≥ 5/h	PSG

*CS, cross sectional; C, controlled; U, uncontrolled; CO, consecutive; P, prospective; R, retrospective; OSA, obstructive sleep apnea; CSA, central sleep apnea; AHI, apnea hypopnea index (events/h); CAI, central apnea index*.

### Central Hypersomnias

The second main group of SDs in patients with CP is represented by central hypersomnias, which can be recognized on PSG. Central hypersomnias are characterized by severe EDS, despite normal quality and timing of nocturnal sleep. ICSD-3 distinguishes three main subtypes: narcolepsy type 1, narcolepsy type 2, and idiopathic hypersomnia (19). Secondary narcolepsy and cataplexy are rare disorders described as a consequence of lesions of lateral hypothalamus orexinergic neurons (13, 24, 26, 39, 51–54). Narcolepsy is rare and characterized by EDS and REM sleep dysregulation manifesting as sleep paralysis, cataplexy, and hypnagogic and hypnopompic hallucinations (19). The diagnosis is based on clinical symptoms and a mean sleep latency of ≤ 8 min with two or more sleep onset REM periods (SOREMPs) on a Multiple Sleep Latency Test (MSLT) or low hypocretin-1 concentration in the cerebrospinal fluid (CSF) (≤110 pg/ml) (19). Cataplexy is defined as more than one episode of generally brief (<2 min), usually sudden bilateral and symmetrical loss of muscle tone with retained consciousness. The episodes are induced by strong emotions, usually positive, with almost all patients reporting some episodes induced by emotions associated with laughter (19). Narcolepsy is strongly associated with obesity (24) and other SDs like REM sleep behavior disorders (55), periodic leg movements during sleep and restless leg sleep syndrome (56), and OSA (57). Jacola et al. (31) assessed EDS in a pediatric CP cohort using the Modified Epworth Sleepiness Scale (M-ESS) and MSLT. M-ESS is a quick screening tool derived from ESS to identify EDS in children by assessing their likelihood to fall asleep in different everyday situations (from 0, which is low probability to fall asleep, to 3, which is high probability to fall asleep). A cutoff of 10 is indicative for EDS (58). EDS was identified by M-ESS in 76% of pediatric CP and strictly related to hypothalamic involvement (31). However, M-ESS may not be sensitive enough to screen pediatric patients for EDS (58), so complete sleep evaluation is often recommended (25). In survivors of childhood brain tumors, hypersomnia/narcolepsy was diagnosed on a median of 6.1 years from diagnosis and 4.7 years from cranial irradiation, and tumor location and radiation therapy were potential risk factors (26). Stimulants improved wakefulness and school performance (26). Table 2 summarizes central hypersomnias and EDS observed in patients with CP.

**Table 2:** Excessive daytime somnolence and secondary narcolepsy in patients with craniopharyngioma.

**References**	**Patients (*n*)**	**SD**	**Study design**	**Prevalence**	**Age**	**Diagnostic criteria**	**Diagnostic tools**
Snow et al. (23)	5 (3 CF)	Daytime sleepiness	C P	5/5 (100%)	11–15 yrs	ESS > 12	ESS, MSLT
Poretti et al. (59)	21	Daytime sleepiness	P U	6/21 (28.5%)	Pediatric	ESS > 10	ESS
Müller et al. (60)	79	Daytime sleepiness	C P	28/79 (35.4%)	Pediatric and Adult	ESS > 10	ESS
van der Klaauw et al. (61)	27	Daytime sleepiness	C P	9/27 (33%)	Adult	ESS > 10	ESS
Lipton et al. (29)	3	Daytime Sleepiness	C (selected hypersomnolent patients)	3/42 (7.14%)	17–22 yrs	Self-Reported	Actigraphy
Crowley et al. (6)	28	Daytime Sleepiness	C P (obese CP vs. obese controls)	20/28 (71.4%)	16–67 yrs	ESS > 10	ESS
Manley et al. (11)	28	Daytime Sleepiness	R U	19/28 (67.8%)	Pediatric and Adult	Self-Reported	Self-Reported
Mandrell et al. (13)	110	Hypersomnia	CS CO U	39/86 (45.3%)	Pediatric and Adult	Tanner Prepubescent MSL ≤ 15; Tanner pubescent MSL ≤ 10	PSG; MSLT
		Narcolepsy		30/86 (34.8%)		Tanner Prepubescent MSL ≤ 15; Tanner pubescent MSL ≤ 10 AND ≥ 2 SOREMPs	PSG; MSLT

*CS, cross sectional; C, controlled; U, uncontrolled; P, prospective; CO, consecutive; R, retrospective; ESS, Epworth sleepiness scale; PSG, polysomnography; MSLT, multiple sleep latency test; SOREMPs, sleep onset REM periods*.

### Circadian Rhythm Sleep-Wake Disorders

CRSWDs are characterized by alterations of the circadian time-keeping system or misalignment of the endogenous circadian rhythm and the external environment (19), associated with sleep-wake disturbances (EDS or insomnia) and distress (19). ICSD-3 distinguishes different types of CRSWDs: delayed sleep-wake phase disorder, advanced sleep-wake phase disorder, irregular sleep-wake rhythm disorder, non-24-h sleep-wake rhythm disorder, shift work disorder, jet lag disorder, and circadian sleep-wake disorder not otherwise specified (19). Patients with CP show frequent disruption of sleep-wake cycle and circadian rhythm (20, 27–30), typically caused by involvement of the hypothalamic SCN and alterations in melatonin transmission (62, 63). Melatonin can be measured in the peripheral blood to diagnose clock disruption, but this requires serial blood samples, and several variables may interfere with correct result interpretation (33), preventing routine use in clinical practice. Sleep logs, prolonged sleep-wake cycle monitoring by actigraphy, and circadian variations of salivary melatonin by dim-light melatonin onset are useful tools to establish a CRSWD diagnosis (64). Patients with CP lacking midnight melatonin peak had impaired sleep quality, increased EDS, and more general and mental fatigue (30). Obesity and EDS were also linked to low midnight-morning melatonin concentration (20). In addition, sleep fragmentation and EDS are frequently reported in CP with a circadian profile characterized by early morning awakening, followed by napping during the afternoon (11, 30, 58). Actigraphy may help to recognize different patterns of SDs and is a reliable tool to estimate total sleep time, sleep latency, sleep onset latency, sleep efficiency, awakenings, and wakefulness after sleep onset (65). Different hypothalamic lesions in obese children with CP may induce shorter (7) or longer sleep onset latency (30). The main CRSWDs in patients with CP are reported in Table 3.

**Table 3:** Sleep-wake cycle alterations in patients with craniopharyngioma.

**References**		**Patients (*n*)**	**Study design**	**Prevalence**	**Age**	**Sleep findings**	**Diagnostic tools**
Lipton et al. (29)	Sleep-Wake cycle alterations Melatonin deficiency	3	C (selected hypersomnolent patients)	3/3 mild OSA	17–22 yrs	(1) Irregular bed time; (2) Frequent night-time activity; (3) Inappropriate daytime episodes of rest; (4) Low melatonin level in patients compared to controls	Actigraphy and Melatonin plasma dosage vs. levels in Historical controls
Pickering et al. (30)	Melatonin deficiency Increased sleep Latency	15	C	Normal melatonin profile and no sleep alterations (6/14) Absent mid night peak of melatonin and impaired sleep quality; EDS and fatigue (6/14) Phase-shifted peak and no sleep alterations (2/14)	18–70 yrs	(1) Unchanged sleep onset (2) Wake up 1 h earlier (3) Higher global score in PSQI (impaired sleep quality, increased sleep latency and increased daytime dysfunction) (4) Lower melatonin (5) Low midnight melatonin associated with increased daytime sleepiness	Sleep Log, PSQI, ESS, SF-36, MFI Saliva melatonin dosage, blood cortisol dosage

*C, controlled; OSA, obstructive sleep apnea; EDS, excessive daytime somnolence; PSQI, Pittsburgh sleep quality index; ESS, Epworth sleepiness scale; SF-36, short form health survey; MFI, multidimensional fatigue inventory*.

## Diagnosis of Sleep Disorders in Patients With CP

In order to appropriately evaluate patients with CP for SDs, it is crucial to investigate suggesting symptoms such EDS, non-restorative sleep, and fatigue. Physicians should obtain a comprehensive clinical history on sleep behaviors, sleep hygiene from patients, and bed partners, even in the absence of evident sleep complaints. Open questions regarding non-restorative sleep, history of observed snoring, apneas, obesity, and EDS should raise the suspicion of comorbid OSA and lead to further investigations. Special care should be paid to inattention, hyperactivity, high blood pressure, enuresis, and failure to thrive that are commonly reported in pediatric OSA (44). In addition, some questionnaires may help to suspect SBDs, EDS, and CRSWDs. Although specific guidelines for SDs in CP and other suprasellar tumors are lacking, the general indications for diagnostic tests should be applied based on clinical suspicion: HSAT for SBDs/OSA, PSG for hypersomnia and MSLT for narcolepsy and other central hypersomnias, and actigraphy for suspected SDs and more specifically CRSWDs and insomnia (19).

**Figure 1:**
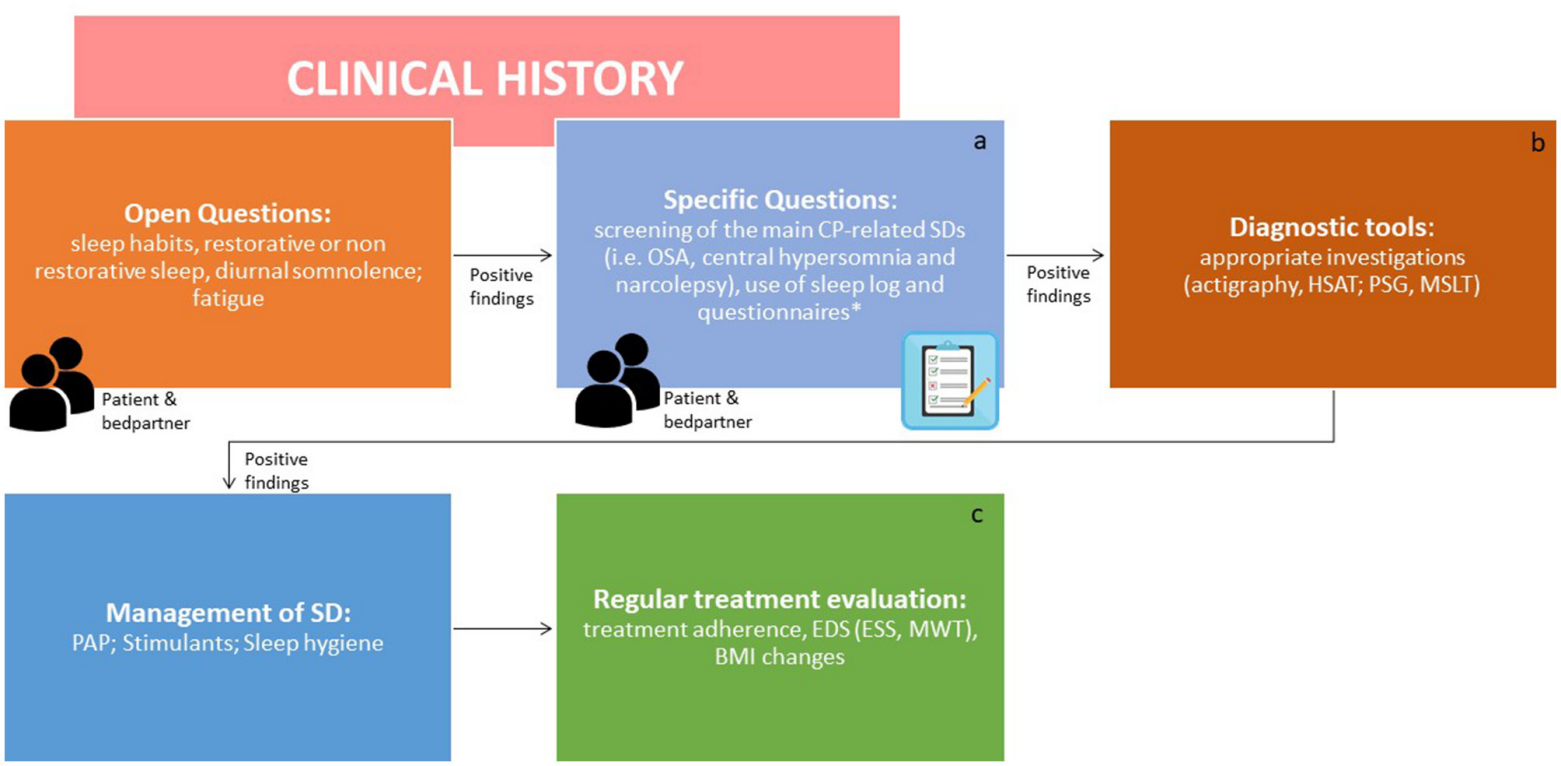
Algorithm for the screening and identification of sleep disorders in patients with craniopharyngiomas. ICSD-3, International classification of sleep disorders version 3; CP, craniopharyngioma; SDs, sleep disorders; OSAS, obstructive sleep apnea syndrome; HSAT, home sleep apnea test; PSG, polysomnography; MSLT, multiple sleep latency test; CRSWD, circadian rhythm sleep-wake disorders. Endocrinologists and neurosurgeons should be involved in the screening step. The second step should be performed by a sleep specialist. ^a^Diaries can help to obtain clinical points in a standardized manner. *The use of formal screening questionnaires for sleep disorders is advisable [i.e., STOP BANG for sleep apnea, Pittsburgh Sleep Questionnaire Index (PSQI) for SDs, Epworth Sleepiness Scale for EDS, Morningness-Eveningness Questionnaire to identify chronotype]. ^b^SDs should be managed as per overall guidelines. ^c^The effects of treatments should be regularly evaluated [adherence to PAP, EDS by ESS score or Maintenance Wakefulness Test (MWT) together with multidisciplinary evaluation of obesity and related cardiometabolic complications as well as appropriate hormone replacement, where present. In particular, body mass index (BMI) should be noticed at each visit].

**Figure 2:**
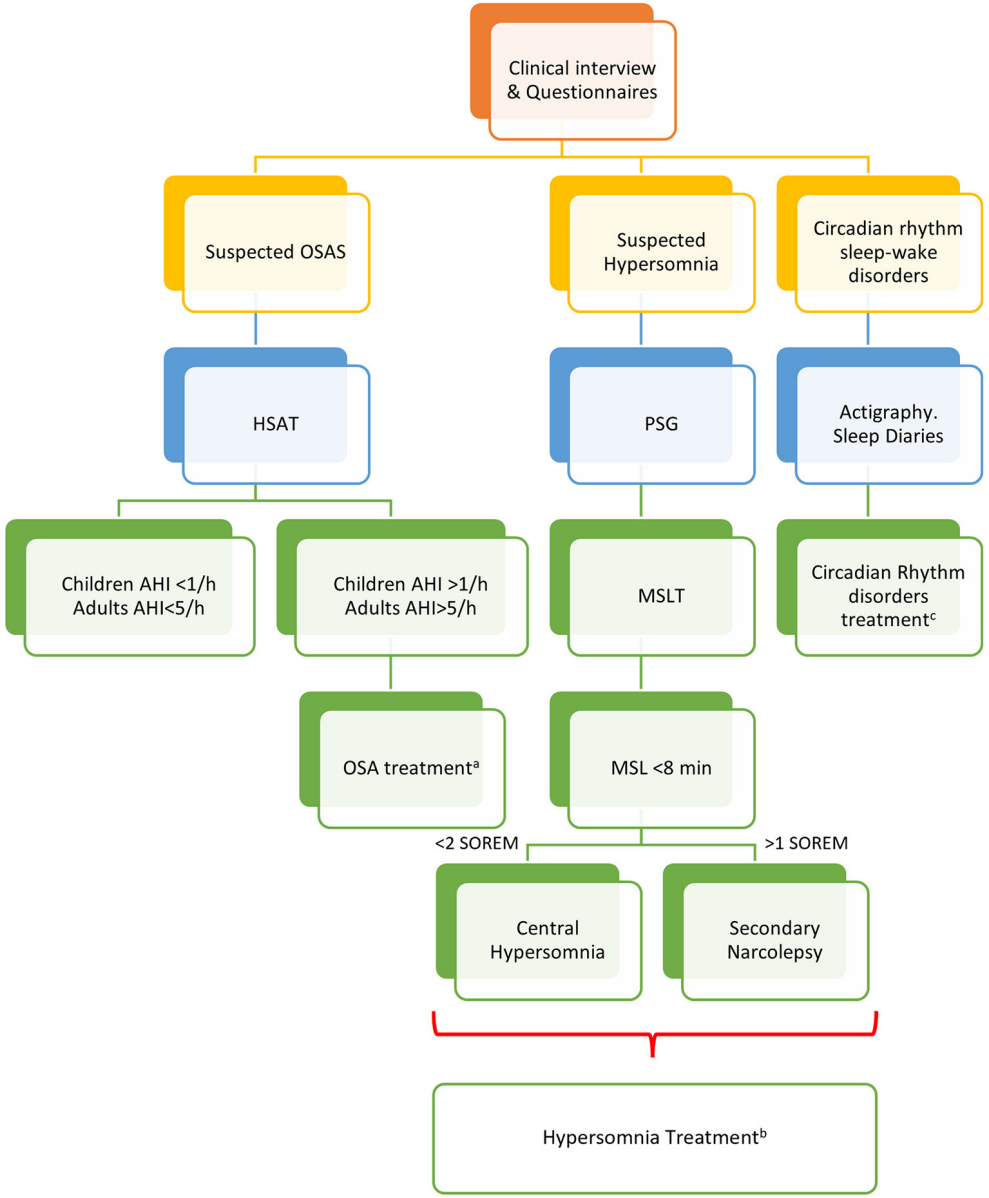
Algorithm for the management of sleep disorders in patients with CP. PSG, polysomnography; OSAS, Obstructive Sleep Apnea (OSA) syndrome; HSAT, home sleep apnea test; AHI, apnea-hypopnea index per hour of sleep; MSLT, multiple sleep latency test; MSL, mean sleep latency; ^a^The management of OSA should include weight loss, avoidance of alcoholic intake and smoking, sleep hygiene, and positional therapy. Positive Airway Pressure (PAP) is considered first-line treatment. Oral appliances may be suggested for mild to moderate OSA and surgery to correct anatomic obstructions (66). ^b^The treatment of central hypersomnias and secondary narcolepsy should include cognitive behavioral therapy (CBT) and approved stimulants (i.e., modafinil, pitolisant, solriamfetol, and sodium oxybate) (67). ^c^Sleep hygiene, CBT, and short-term pharmacologic approach should be considered for insomnia and CRSWD (68).

**Figure 3:**
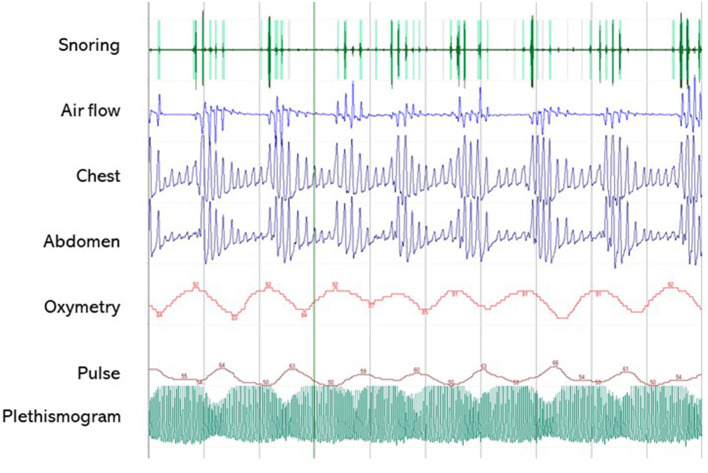
A 5-min segment from home sleep apnea test (HSAT) in the diagnosis of sleep-related breath disorders in a 51-year old male patient with CP. The patient was operated on for a huge supra- and retrosellar craniopharyngioma with hydrocephalus and ataxia, achieving complete resection of an adamantinomatous lesion. He developed post-operative diabetes insipidus and partial hypopituitarism, and had severe weight gain (+50 kg) with snoring and markedly excessive daytime somnolence (EDS), confirmed by a high ESS score (16/24). HSAT confirmed the presence of severe OSA syndrome (AHI 58.8/h), characterized by several obstructive apneas. PAP treatment induced the disappearance of EDS (ESS score 7/24). Overall, the patient was very compliant to lifestyle interventions and endocrinological management, and significant weight loss (−30 kg) was also achieved.

**Figure 4:**
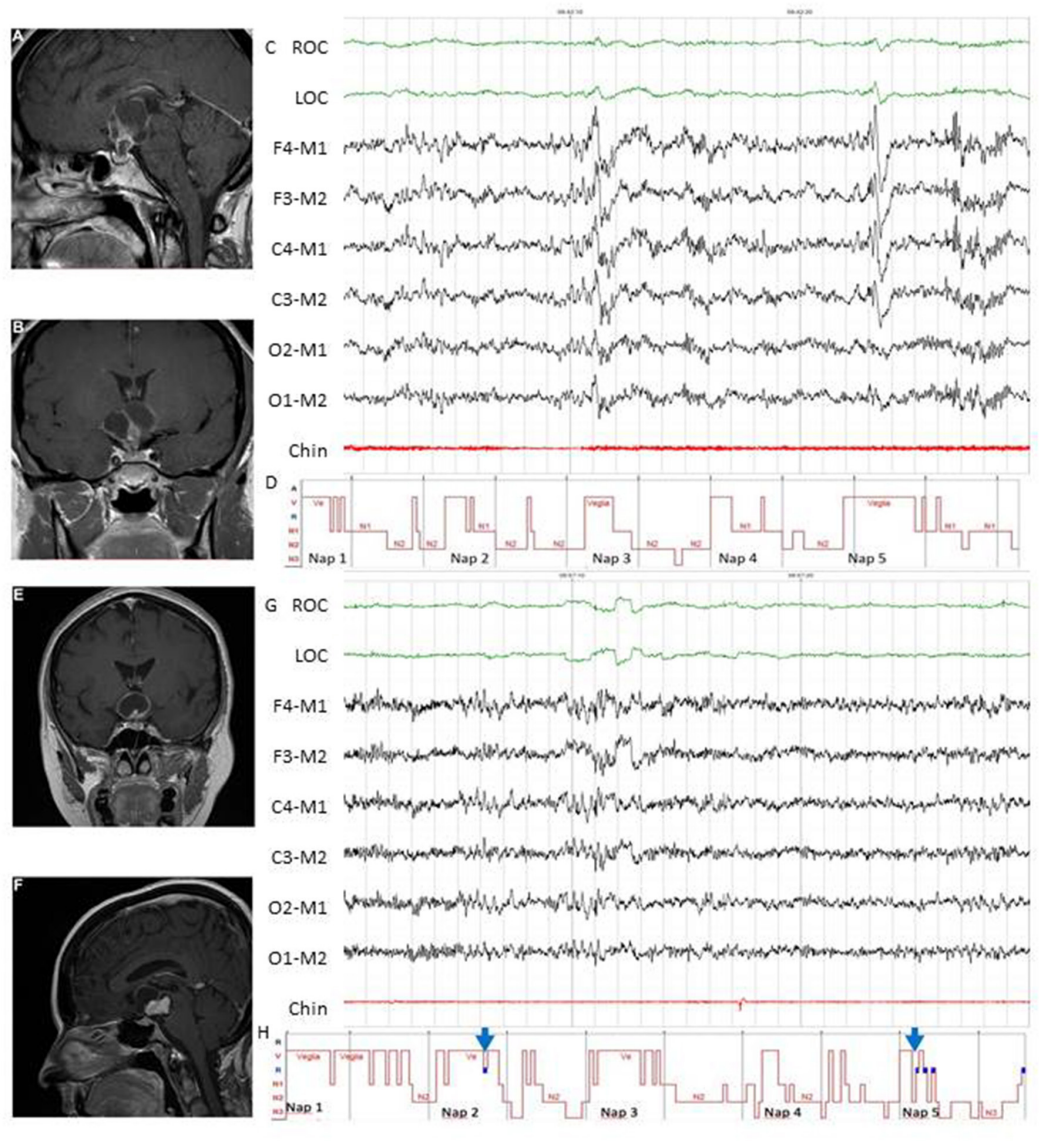
Examples of MSLT in the diagnosis of central hypersomnias in two female patients with CP. Patient 1 **(A–D)**. Pre-operative evaluation of a 44-year-old woman who presented with spontaneous hypothalamic syndrome with severe weight gain (+30 kg) associated with headache, secondary amenorrhea, asthenia, insomnia, and diurnal somnolence. Contrast-enhanced T1-weighted magnetic resonance imaging (MRI) revealed a huge solid and cystic suprasellar lesion [**(A)**, coronal view] with posterior extension [**(B)**, sagittal view]. MSLT showed a 30-s epoch of NREM sleep (N2) **(C)** with hypnogram confirming severe excessive daytime somnolence (mean sleep latency 4.2 min) without sleep-onset REM in 5 of 5 nap periods **(D)**. A diagnosis of central hypersomnia was made. Patient 2 **(E–H)**. Post-operative evaluation of a 52-year-old woman affected by complex post-operative sleep disorders accompanied by diabetes insipidus, pan-hypopituitarism, ongoing severe weight gain (+7 kg before surgery, +30 kg after surgery) and asthenia. Preoperative contrast-enhanced T1-weighted MRI showed a huge solid and cystic suprasellar lesion [**(E)**, coronal view] with posterior extension [**(F)**, sagittal view]. Excessive daytime somnolence persisted on continuous PAP for documented post-operative OSA (data not shown), and MSLT was recently proposed. The MSLT showed a 30-s epoch of REM sleep **(G)**, with hypnogram confirming severe excessive daytime somnolence (mean sleep latency 2.3 min) with sleep-onset REM in 2 of 5 nap periods [**(H)**, see blue arrows]. A diagnosis of secondary narcolepsy was made, and a stimulant oral agent (modafinil) was started. In both patients, complete tumor resection was achieved, and pathological examination revealed adamantinomatous (patient 1) and papillary (patient 2) craniopharyngiomas. ROC, right oculogram; LOC, left oculogram; M1 and M2 reference electrodes placed on the mastoid process; Chin, Chin electromyogram.

**Figure 5:**
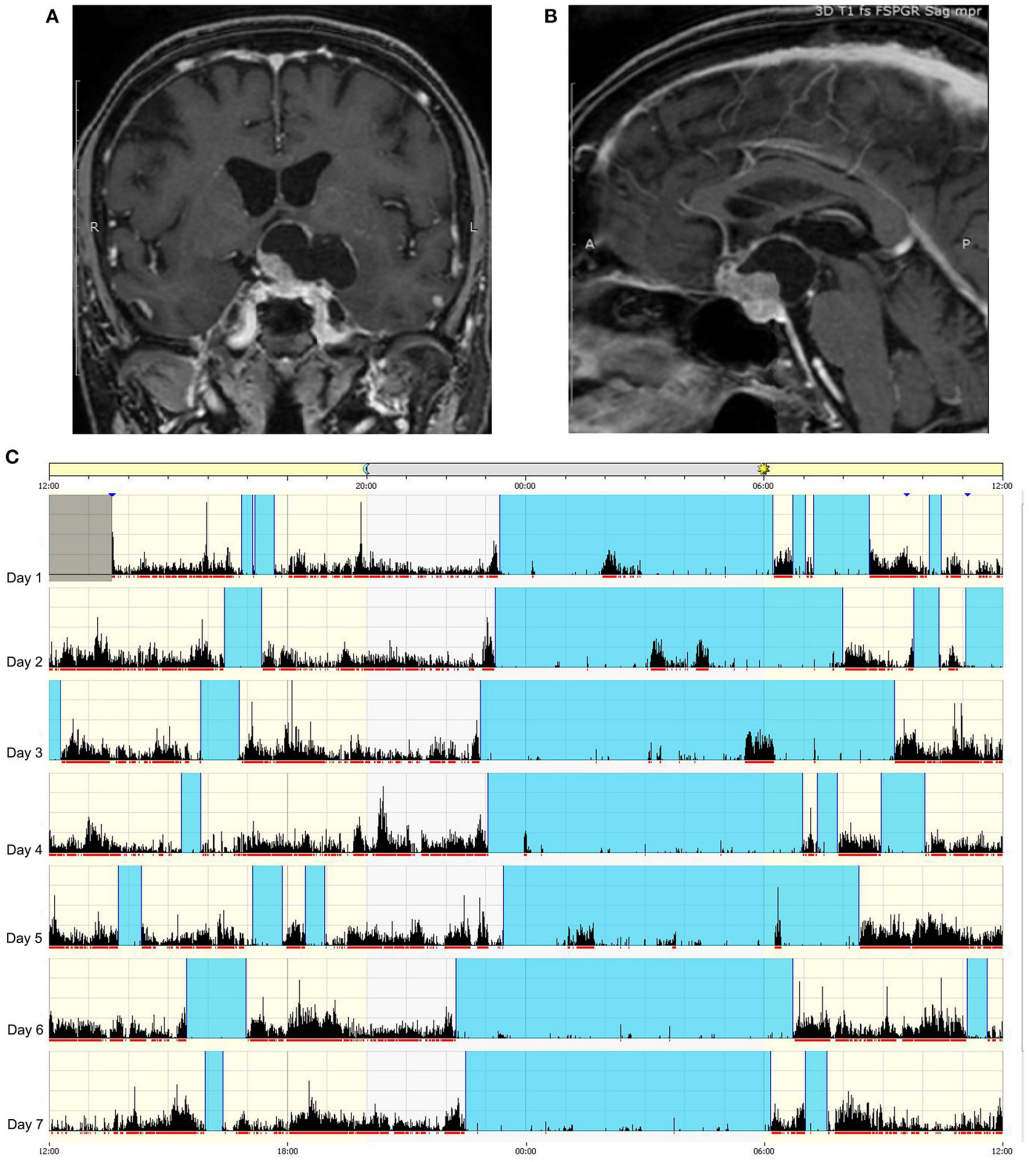
Example of circadian sleep-wake alteration evaluation by actigraphy. A 75-year-old female patient came to our observation because of headache and visual loss in the context of recent and rapidly worsening neurological symptoms consisting of insomnia, excessive daytime somnolence, cognitive impairment, reduced appetite, and weight loss. No poliurodyspia was present, and basal pituitary function and electrolytes were normal. Contrast-enhanced T1-weighted MRI revealed a mixed cystic and solid tumor consistent with suprasellar craniopharyngioma **(A)** with retrosellar extension **(B)**. Sleep-wake patterns are displayed for individual days on actigraphy **(C)**: vertical black bars and the red line under each day indicate movement, and the absence of black bars indicates supposed sleeping periods. The blue band designates the sleep period. The actigram shows frequent nighttime activity, severe insomnia, sleep fragmentation, and frequent short diurnal naps. The patient is currently awaiting surgery.

A clinical algorithm aimed to suspect SDs in patients with CP and summarize their approach and follow-up according to current sleep medicine guidelines (19) is proposed in Figure 1. Based on clinical suspicion, another algorithm is proposed in Figure 2 to guide the diagnosis and treatment of the most frequently reported SDs. Examples of OSA, central hypersomnia and CRSWD observed in adult patients with CP in our institution and diagnosed by HSAT, PSG and actigraphy, respectively, are shown in Figures 3–5. Of note, all these patients had a supra- and retro-sellar extension at pre-operative magnetic resonance imaging (MRI). Table 4 provides a glossary and abbreviation list of sleep terms reported in our study, and Table 5 reports the current definition and diagnostic criteria of the most relevant SDs reported in patients with CP.

**Table 4:** Glossary of sleep terms included in the review.

**Term**	**Abbreviation**	**Definitions**
Actigraphy		A non-invasive technique that measures physical activity levels of a subject by means of a wristwatch-like motion-sensing device that can be worn for prolonged periods of time. Its use is considered useful to diagnose CRSWDs, insomnia and other sleep disorders (i.e., OSA, restless legs syndrome)
Apnea-Hypopnea index	AHI	A diagnostic tool for determining the presence and severity of OSA. It represents the average number of apneas and hypopneas by hour during sleep
Circadian rhythm sleep wake disorders	CRSWDs	Chronic or recurrent patterns of sleep-wake rhythm disruption primarily caused by an alteration in the endogenous circadian timing system or misalignment between the endogenous circadian rhythm and the sleep-wake schedule
Cognitive behavioral therapy of insomnia	CBTi	A short, structured, and evidence-based approach to improve symptoms of insomnia, by identifying and replacing thoughts and behaviors that cause or worsen sleep problems
Epworth sleepiness scale	ESS	A subjective questionnaire to measure daytime sleepiness in the past month
Home sleep apnea test	HSAT	An alternative simplified medical test for the diagnosis of OSA in uncomplicated adults presenting with signs and symptoms that indicate an increased risk of moderate to severe OSA. It does not include electroencephalography, electrooculogram, and electromyography
Maintenance wakefulness test	MWT	An objective measure of daytime vigilance that is used to quantify changes in the ability to stay awake
Morningness–Eveningness questionnaire	MEQ	A self-assessment tool that can provide details regarding an individual's subjective timing preferences
Multiple sleep latency test	MSLT	An objective measure of daytime sleepiness that is used to measure physiological sleep tendency in the absence of alerting factors among 5 diurnal naps. MSL (mean sleep latency) is the mean of each sleep latency
Obstructive sleep apnea	OSA	Obstructive sleep apnea (OSA) is a sleep-related breathing disorder that involves a decrease or complete halt in airflow despite an ongoing effort to breathe
Pittsburgh sleep quality index	PSQI	A self-rated, subjective, questionnaire to evaluate sleep quality, and disturbances over a 1-month time interval
Polysomnography	PSG	A comprehensive sleep study including electroencephalography, electrooculogram, chin and leg electromyography, body position, airflow, respiratory movement, oxygen saturation. PSG is considered the “gold standard” of sleep study
Positive airway pressure	PAP	PAP is the first-choice treatment for OSA involving devices to maintain upper airway patency by increasing the upper airway pressure
Sleep onset REM periods	SOREMPs	REM sleep period occurring ≤15 min after the onset of sleep on an overnight PSG or MSLT
Stop-BANG questionnaire	Stop-BANG	An easy to use, concise, effective, and reliable OSA screening tool including **S**noring, **T**iredness, **O**bserved a**P**nea, high **B**P, BMI, **A**ge, **N**eck circumference, and male **G**ender

**Table 5:** Classification and definition of sleep disorders of interest in patients with craniopharyngioma (19).

**Term (abbreviation)**	**Definition**	**Diagnostic criteria (ICSD-3)**
Central disorders of hypersomnolence	A group of disorders in which the primary complaint is daytime sleepiness not caused by disturbed nocturnal sleep or misaligned circadian rhythms. Other sleep disorders may be present, but they must be adequately treated before a diagnosis in this category can be established. This group includes (a) Narcolepsy type 1 (b) Narcolepsy type 2 (c) Idiopathic hypersomnia (d) Kleine-Levin syndrome (e) Hypersomnia due to a medical disorder (f) Hypersomnia due to a medication or substance (g) Hypersomnia associated with a psychiatric disorder (h) Insufficient sleep syndrome	**Narcolepsy type 1** **Criteria A and B must be met** A. The patient has daily periods of irrepressible need to sleep or daytime lapses into sleep occurring for at least 3 months B. The presence of one or both of the following: 1. Cataplexy (defined as more than one episode of generally brief (<2 min), usually bilaterally symmetrical, sudden loss of muscle tone with retained consciousness) and a mean sleep latency of ≤ 8 min and two or more sleep-onset REM periods (SOREMPs) on an MSLT performed according to standard techniques. A SOREMP (within 15 min of sleep onset) on the preceding nocturnal polysomnogram may replace one of the SOREMPs on the MSLT 2. CSF hypocretin-1 concentration, measured by immunoreactivity, is either ≤110 pg/mLor <1/3 of mean values obtained in normal subjects with the same standardized assay **Narcolepsy type 2** **Criteria A and E must be met** A. The patient has daily periods of irrepressible need to sleep or daytime lapses into sleep occurring for at least 3 months. B. A mean sleep latency of ≤8 min and two or more sleep-onset REM periods (SOREMPs) are found on a MSLT performed according to standard techniques. A SOREMP (within 15 min of sleep onset) on the preceding nocturnal polysomnogram may replace one of the SOREMPs on the MSLT C. Cataplexy is absent D. Either CSF hypocretin-1 concentration has not been measured or CSF hypocretin-1 concentration measured by immunoreactivity is either >110 pg/mL or >1/3 of mean values obtained in normal subjects with the same standardized assay E. The hypersomnolence and/or MSLT findings are not explained more clearly by other causes such as insufficient sleep, obstructive sleep apnea, delayed sleep phase disorder or the effect of medication or substances or their withdrawal **Hypersomnia due to medical disorders Criteria A–D must be met** A. The patient has daily periods of irrepressible need to sleep or daytime lapses into sleep occurring for at least 3 months B. The daytime sleepiness occurs as a consequence of a significant underlying medical or neurological condition C. If an MSLT is performed, the mean sleep latency is ≤ 8 min, and fewer than two sleep onset REM periods (SOREMPs) are observed D. The symptoms are not better explained by another untreated sleep disorder, a mental disorder, or the effects of medications or drugs. (a) If criteria for narcolepsy are fulfilled, a diagnosis of narcolepsy type 1 or type 2 due to a medical condition should be used rather than hypersomnia due to a medical condition; (b) In patients with severe neurological or medical disorders in whom it is not possible or desirable to perform sleep studies, the diagnosis can be made by clinical criteria
Circadian rhythm sleep wake disorders (CRSWDs)	Chronic or recurrent patterns of sleep-wake rhythm disruption primarily caused by an alteration in the endogenous circadian timing system or misalignment between the endogenous circadian rhythm and the sleep-wake schedule. This group includes 1. Delayed sleep–wake phase disorder; 2. Advanced sleep–wake phase disorder; 3. Irregular sleep–wake rhythm disorder; 4. Non-24 h sleep-wake rhythm disorder; 5. Shift work disorder; 6. Jet lag disorder; 7. Circadian sleep–wake disorder not otherwise specified	**General criteria for circadian rhythm sleep–wake disorder Criteria A–C must be met** A. A chronic or recurrent pattern of sleep–wake rhythm disruption due primarily to alteration of the endogenous circadian timing system or misalignment between the endogenous circadian rhythm and the sleep–wake schedule desired or required by an individual's physical environment or social/work schedules B. The circadian rhythm disruption leads to insomnia symptoms, excessive sleepiness or both C. The sleep and wake disturbances cause clinically significant distress or impairment in mental, physical, social, occupational, educational, or other important areas of functioning
Insomnia	A persistent difficulty with sleep initiation, duration, consolidation, or quality that occurs despite adequate opportunity and circumstances for sleep, and results in some form of daytime impairment	**Chronic Insomnia** **Criteria A–F must be met** **A. The patient reports, or the patient's parent or caregiver observes, one or more of the following**: 1. Difficulty initiating sleep
		2. Difficulty maintaining sleep 3. Waking up earlier than desired 4. Resistance to going to bed on appropriate schedule 5. Difficulty sleeping without parent or caregiver intervention **B. The patient reports, or the patient's parent or caregiver observes, one or more of the following related to the nighttime sleep difficulty**: 1. Fatigue/malaise 2. Attention, concentration or memory impairment 3. Impaired social, family, occupational, or academic performance 4. Mood disturbance/irritability 5. Daytime sleepiness 6. Behavioral problems (e.g., hyperactivity, impulsivity, aggression) 7. Reduced motivation/energy/initiative 8. Proneness for errors/accidents 9. Concerns about or dissatisfaction with sleep **C. The reported sleep/wake complaints cannot be explained purely by inadequate opportunity (i.e., enough time is allotted for sleep) or inadequate circumstances (i.e., the environment is safe, dark, quiet, and comfortable) for sleep** **D. The sleep disturbance and associated daytime symptoms occur at least three times per week E. The sleep disturbance and associated daytime symptoms have been present for at least 3 months** **F. The sleep/wake difficulty is not explained more clearly by another sleep disorder**
Sleep-Related breathing disorders (SDBs)	A range of conditions characterized by abnormal breathing during sleep; in many cases this is associated with narrowing or obstruction of the upper airway (pharynx). The disordered breathing ranges from intermittent, partial obstruction of the airway without sleep disturbance (snoring) to frequent apneas associated with repetitive hypoxaemia and arousals leading to sleep disruption and daytime sleepiness. This group includes obstructive sleep apnea (OSA) syndrome, central sleep apnea disorders, sleep-related hypoventilation disorders and sleep-related hypoxaemia disorders. OSA is a sleep disorder involving cessation or significant decrease in airflow in the presence of breathing effort	**OSA (ADULT)** **(A and B) or C satisfy the criteria** **A. The presence of one or more of the following**: The patient complains of sleepiness, non-restorative sleep, fatigue or insomnia symptoms The patient wakes with breath holding, gasping or choking The bed partner or other observer reports habitual snoring, breathing interruptions or both during the patient's sleep The patient has been diagnosed with hypertension, a mood disorder, cognitive dysfunction, coronary artery disease, stroke, congestive heart failure, atrial fibrillation, or type 2 diabetes mellitus **B. Polysomnography (PSG) or HSAT (Home Sleep Apnea Test) demonstrates:** Five or more predominantly obstructive respiratory events [obstructive and mixed apneas, hypopneas or respiratory effort-related arousals (RERAs)] per hour of sleep during a PSG or per hour of monitoring (HSAT) or **C. PSG or HSAT demonstrates:** Fifteen or more predominantly obstructive respiratory events (apneas, hypopnoeas, or RERAs) per hour of sleep during a PSG or per hour of monitoring (HSAT)
		**OSA (PEDIATRIC)**
		**Criteria A and B must be met** **The presence of one or more of the following**: 1. Snoring 2. Labored, paradoxical, or obstructed breathing during the child's sleep 3. Sleepiness, hyperactivity, behavioral problems, or learning problems **PSG demonstrates one or more of the following**: 1. One or more obstructive apneas, mixed apneas, or hypopneas, per hour of sleep 2. A pattern of obstructive hypoventilation, defined as at least 25% of total sleep time with hypercapnia (PaCO2 > 50 mm Hg) in association with one or more of the following: (a) Snoring, (b) Flattening of the inspiratory nasal pressure waveform, (c) Paradoxical thoracoabdominal motion

*ICSD-3: International Classification of Sleep Disorders - Third Edition (19)*.

## Prevention and Treatment of Sleep Disorders in Patients With Craniopharyngioma

A multidisciplinary approach is crucial to target several factors involved in the onset and progression of SDs in patients with CP. The choice of a safe neurosurgical approach is the first step in the prevention of hypothalamic damage, and optimization of hormone replacement therapy is necessary in patients with hypopituitarism and/or DI to correct their potential contribution to SDs. In the presence of HS, complementary strategies should be put in place to simultaneously address sleep health (direct strategies) and HO (indirect strategies). We will, therefore, analyze risk factors for the development of HS and SDs, and focus on their prevention and treatment, pointing out the potential benefits of sleep medicine in such patients.

### Neurosurgical Treatment

The optimal treatment of patients with CP is still a matter of debate due to difficulty in finding effective balance between an aggressive approach aiming for complete resection to prevent recurrences, and a more conservative approach aiming to reduce the risk of post-operative complications and long-term sequelae. The surgical approach itself has also significantly evolved in the last decades. Controversies about surgical objectives and techniques are related to the complexity of the anatomical location and extension of CP, which may arise anywhere along the craniopharyngeal duct. Nearly 95% have a suprasellar component, up to 75% are intra-suprasellar and only a minority are purely intrasellar (<10%) (1). Suprasellar CPs may occasionally extend to the anterior, middle, or posterior fossa, rarely they are completely situated within the 3rd ventricle, and hydrocephalus may be present more frequently in children than in adults (1). Radical resection has long been considered as the therapy of choice at any age for the primary treatment of CP, and several open transcranial microsurgical (TC) approaches have been developed, offering uni- or bilateral access to the tumor. TC surgery allows for good maneuverability but requires some degree of brain retraction and optic nerve and vascular manipulation, and increased resection rates have been associated with increased morbidity and mortality, in particular with neuroendocrine dysfunction and HO (1–3). Exposure of infra-chiasmatic, retrosellar, and interpeduncular extension of some midline tumors may also be limited. Conversely, microsurgical transsphenoidal approaches provide limited exposure and maneuverability in the suprasellar space and may cause CSF leak. In the last 15 years, the development of expanded endoscopic endonasal approaches (EEAs) in skull base surgery has changed the approach to CP (69–72). Although initially limited to resection of intrasellar tumors, with increasing experience and improved technology, EEAs are being increasingly used for suprasellar CP, and the incidence of CSF leaks has been reduced by the use of multilayer reconstruction techniques (69, 70). In experienced hands, EEAs may now be proposed for the treatment of midline infra-chiasmatic and suprasellar CP, and, in some cases extended to CP of the 3rd ventricle (70). A recent consensus statement of the European Association of Neurosurgical Societies (EANS), skull base section, recommends surgery in tertiary referral centers and further supports the role of EEAs as suitable to most adult CP (71). For anatomical reasons, endonasal surgery has been considered difficult in the pediatric population, but the place of EAAs in the treatment of pediatric CP may progressively increase with improved technology (73).

Preoperative neuroradiological imaging is essential for the diagnosis and surgical treatment of CPs, and their characteristics have been well-described (74). Adamantinomatous CP typically present as multilobulated mixed solid/cystic intra/suprasellar masses, with frequent calcifications (90%) on X-ray or computerized tomography (CT). The solid component is unevenly hypointense in T2 on MRI and, together with the peripheral component of the cysts, shows irregular contrast enhancement. Cystic components are hypointense on T1, and their intensity in T2 depends on their protein content. Papillary CPs are suprasellar and devoid of calcifications. Of note, perilesional edema in FLAIR may hardly be distinguished from the lesion when it infiltrates the chiasm, hypothalamus, or mammillary bodies. Identifying the hypothalamus and mammillary bodies is essential, although controversies remain about the relative impact of pre-operative hypothalamic involvement (HI) itself and surgical strategies on long-term post-operative outcome, including post-operative HO. Sainte-Rose et al. (75), Van Gompel et al. (76), and Muller et al. (77) have proposed different neuroradiological classifications of CP to define the grading of HI. The prognostic value of HI according to either classification, together with additional characteristics such as unidentified pituitary stalk, retrochiasmatic extension, and peri-tumoral edema, has been confirmed in a multifactorial analysis of risk factors for the development of HS and morbid HO (78).

Differences in CP management worldwide and across the last decades have been discussed in details elsewhere (2). As a general rule, experienced neurosurgeons currently advise gross total resection where presumably safe, and conservative approaches with subtotal tumor removal in the presence of risk factors for significant post-operative morbidity. Interestingly, according to a meta-analysis performed on adult patients with CP, conservative surgery itself was associated with reduced risk of post-operative hypopituitarism and DI but did not significantly prevent weight gain (79). Similar findings arise from a pediatric meta-data analysis in the United Kingdom (80), pointing out unresolved issues in the identification of individual risk factors and personalized surgery. An algorithm for risk-adapted hypothalamic-sparing surgery (HSS) was first proposed by Sainte-Rose and Puget in pediatric CP, limiting the indications for extensive surgical resection (ESR) to CP presenting with no HI or with hypothalamic compression without invasion (75). In their experience, patients receiving risk-adapted HSS had similar relapse and progression rates, with a significant benefit in weight gain, when compared with their historical cohort of patients with CP treated by ESR (morbid obesity 28 vs. 54% and normal post-operative BMI at last follow-up 38 vs. 17%, respectively) (81). Although validation of this single-center observation in a prospective multicenter setting is still missing, additional studies have been conducted to evaluate the outcome of HSS in patients with CP. In a recent retrospective analysis of the German multicenter KRANIOPHARYNGIOMA 2007 cohort, neither pre-operative HI nor anterior hypothalamic surgical injury, but posterior hypothalamic surgical injury was significantly associated with increased risk of obesity and lower QoL (82). Hence, the authors proposed that HSS should particularly point to a “posterior HSS.” However, the potential benefits of less aggressive surgical approaches to CP on SDs have deserved less attention than HO. Compared to TC surgery, EEA was recently reported to be associated with lower incidence of DI, but post-operative weight gain was not significantly lower and still depended on tumor volume and pre-operative BMI (83). SDs were not addressed in a large series of CP managed by HSS (81, 82). However, encouraging results were reported about potential improvement of sleep-wake cycle and body core temperature in a small series of CP of the 3rd ventricle managed by EEA (84). The recent consensus statement on the surgical treatment of adult CP by EANS skull base section suggests to systematically evaluate pre-operative hypothalamic function (regulation of weight, body temperature, and sleep-wake cycles) and encourages further attention to their post-operative evolution in the future (71).

A drawback of conservative approaches to CP is their potential to recur, and multiple recurrences are a serious concern after incomplete tumor resection, especially in children. The best results in terms of progression-free survival after conservative surgery have been reported in association with post-operative radiotherapy and are similar to those obtained by gross total resection. However, side effects of irradiation are delayed and may include hypothalamic complications (9). Among modern techniques, proton therapy may take an increasing place in the treatment of residual or recurrent CP because of the dosimetric characteristics of protons and limited off-target toxicity (2, 85).

### Endocrine-Metabolic Treatments and Lifestyle Interventions

Although lifestyle and endocrinological/metabolic interventions are not resolutive in patients with HS, they still play a fundamental role in the control of homeostasis, optimization of SD treatment, and prevention of long-term cardio-metabolic/vascular complications. The essential role of short-term post-operative management of endocrine deficiencies and sodium/water imbalance is beyond the scope of this article. It should be remembered, however, that excessive post-operative fluctuations in serum osmolarity due to DI, SIADH, and/or salt wasting syndrome should be avoided, as excessively rapid corrections of hyponatremia may lead to irreversible neurological damage (86, 87). Patients should be clearly informed on the potential risks, clinical manifestations and timing of neuroendocrine alterations and HS following surgery, and the need for long-term endocrinological follow-up. Guidelines on the treatment of single or multiple pituitary deficiencies are currently available (88), and we will focus on the benefits and potential risks of hormone replacement therapy on SDs.

#### Hormone Replacement Therapy

The relationship between hormone replacement and sleep health is complex and bidirectional. Optimal hormone replacement therapy may have a beneficial effect on muscle function, including upper airway dilator muscles, body composition, and metabolism, as well as fatigue and mood. Conversely, overtreatment maybe deleterious for sleep, and worsens OSA or sleep-wake cycle and circadian rhythm. Even in the presence of undamaged hypothalamic-pituitary connections, sleep fragmentation and reduction in slow wave sleep impair circadian pituitary hormone regulation, in particular ACTH and GH (89). Therefore, fine-tuning of hormone replacement is needed to optimize metabolic, cardiovascular, and sleep issues in patients with CP.

##### Diabetes Insipidus

Diabetes insipidus (DI) may be transient or life-long and requires desmopressin replacement therapy according to current guidelines (87, 88). Uncontrolled DI is characterized by polyurodipsia (>50 mL/kg of body weight/24 h) with nycturia and deleterious consequences on sleep quality, increasing daytime sleepiness and fatigue (88, 90). Conversely, overtreatment results in hyponatremia and related neurological complications (86). A minority of CP patients with hypothalamic injury present an impaired sense of thirst, which should be promptly recognized by systematic evaluation of water balance. Adipsia complicates clinical management, reduces QoL (91, 92), and exposes patients to the risk of severe and potentially fatal dehydration (92). Patients and their families should be informed on such risks and the potential recovery of DI to optimize desmopressin treatment.

##### Glucocorticoid Replacement Therapy

Patients affected by either primary or secondary glucocorticoid deficiency complain of fatigue and impaired QoL with reduced daytime activities (93). Despite the risk of acute adrenal insufficiency being lower in patients with corticotroph deficiency because of a typically preserved mineral corticoid function, it may also occur and requires appropriate education of patients and their families. Because any available replacement therapy is unable to reproduce the physiological rhythm of cortisol secretion, patients are frequently exposed to supraphysiological evening levels of cortisol, which may impact on sleep quality, sleep latency, and daytime functioning (93, 94). It may be, therefore, be useful to prefer modified-release hydrocortisone to a standard replacement therapy with twice or trice daily oral hydrocortisone. Modified-release hydrocortisone replacement therapy best approaches physiological cortisol secretion and may have a favorable impact on body weight control, metabolism, immune function, and QoL (95). The single morning administration of modified-release hydrocortisone may also improve patient compliance in the setting of multiple treatments for hormone replacement and/or associated co-morbidities. We, therefore, suggest, where available, to consider modified-released hydrocortisone in the long-term treatment of patients with complex CP, including those with SDs.

##### Thyroid Hormone Replacement Therapy

Thyroid function and sleep also have a bidirectional relationship, influencing each other through the circadian clock (96). Hypothyroidism results in poor sleep quality and architecture (96), and may trigger or worsen preexisting OSA (97). This may occur as a consequence of impaired neural response to hypoxemia and hypercapnia, increased airway resistance due to mucoprotein deposition, increase in BMI, and changes in upper airway muscle activity (96). Conversely, over-replacement may favor insomnia, increase oxygen consumption, and impact on muscle and cardiovascular function as observed in hyperthyroidism (98). Therefore, optimizing thyroxine replacement therapy may contribute to improve sleep quality and architecture in patients with CP.

##### Testosterone Replacement Therapy

Because testosterone replacement therapy has pleiotropic benefits in hypogonadal men, including improvement in fatigue, lean mass, and hemoglobin concentration, it should be considered in hypogonadal male patients with CP according to current guidelines (88). The association between testosterone replacement and OSA is controversial and has not been specifically addressed in patients with CP, but the Endocrine Society recommends against testosterone replacement in patients with severe untreated OSA (99). In a large retrospective study, an elevated risk of OSA among testosterone users compared with controls was observed (100). Potential mechanisms include the impact of androgens on muscle contraction and neuromuscular control of upper airway muscles; increase in oxygen consumption leading to hypoxia, and changes in neural response to hypoxemia and hypercapnia (100). Thus, clinicians should be careful in prescribing testosterone replacement to patients CP and untreated OSA, and reevaluation during ventilation treatment may be useful. Of note, obesity in itself is frequently accompanied by functional hypogonadism. Because the cardiovascular risks and benefits of testosterone replacement are also debated (101), a multidisciplinary evaluation is advisable in patients with complex CP.

##### Recombinant Human Growth Hormone Replacement Therapy

Growth hormone (GH) deficiency is generally the first to occur and the last to be replaced in patients with CP. The diagnosis of severe GH deficiency depends on patient age (childhood, transition, adult) and may be influenced by BMI and the presence and/or replacement of other pituitary deficits (88, 102). GH replacement has a beneficial effect on growth, body composition, and neurophysiological outcome (103), and does not influence the risk of CP relapse (104). Despite GH replacement being suspected to worsen OSA in adults, this remains controversial (105). In adults affected by the Prader Willi syndrome (PWS), a genetic disorder sharing with CP many features of hypothalamic dysfunction, including severe obesity and SDs, GH replacement was found to be safe and did not significantly impair sleep parameters in patients without SDs (106). However, consensus guidelines on PWS recommend to perform PSG before GH replacement and possibly to repeat the test within 3–6 months of treatment, with an ear, nose, and throat evaluation in the presence of OSA (107). Indeed, upper airway obstruction may occur because of GH/IGF1 effects on lymphoid tissue stimulation (107) and sodium/water retention with fibroblast stimulation, leading to soft tissue swelling (106); worsening of OSA in an adolescent patient during GH replacement resolved after tonsillectomy and adenoidectomy (108). GH replacement in adults may be limited by compliance issues and contraindications such as hyperglycemia, and overtreatment should be avoided as it may induce headache and long-term complications as reported in pathological GH/IGF1 excess, including cardiovascular and cardiorespiratory/OSA and neoplastic diseases (88).

#### Lifestyle and Metabolic Interventions

Personalized hypocaloric diet and daily physical activity should always be recommended to target energy expenditure and hyperinsulinemia, and counteract weight gain. Both should be started before surgery in the presence of preexisting obesity or HS and in the early post-operative period if hypothalamic injury is suspected. Several drugs have been proposed to patients affected by HO (1, 5, 109), and an individualized stepwise treatment algorithm has been recently proposed according to predominant clinical complications of HS (5).

The first-line pharmacological treatment of obesity is usually metformin, which increases insulin sensitivity. Metformin was found in a non-diabetic rat model to improve central sleep apnea (110) and some beneficial effects on sleep quality, efficiency, and duration have also been reported in patients with type 2 DM (111, 112). The underlying mechanisms remain unclear. However, as hypoxia increases the risk of lactic acidosis, a rare but severe side effect of metformin, hypoxic patients with CP should be recognized in order to adapt the daily dose of the drug, with prompt discontinuation in the presence of acute respiratory or systemic conditions. Antidiabetic drugs such as glucagon-like 1 (GLP-1) agonists and sodium-glucose contrasporter-2 (SGLT2) inhibitors may be considered to optimize weight loss and metabolic control. The GLP-1 receptor is expressed in several areas of the central nervous system, in particular the hypothalamic arcuate nucleus, where it directly stimulates POMC/CART neurons while indirectly inhibiting NPY/AGRP neurons, thereby increasing satiety and reducing hunger (113). Central effects, therefore, complete the peripheral action of GLP-1 agonists. Among them, exenatide and liraglutide have been used with some benefits in patients with CP (1, 5). Liraglutide 3 mg was studied in non-diabetic obese patients suffering from moderate or severe OSA, in the absence of positive airway pressure (PAP) therapy, and a significant reduction in weight and AHI was observed after 32 weeks of treatment, with a trend toward improved oxygen saturation, sleep architecture, and sleep/health-related QoL outcomes (114). Among SGLT2 inhibitors, empagliflozin has beneficial effects on cardiovascular and renal outcomes in type 2 diabetic patients and may reduce the risk of new-onset OSA (115), possibly mediated by weight loss and the diuretic/natriuretic action of the drug (115). It may, therefore, be attractive in diabetic patients with CP unless fluid and electrolytic imbalance is present, although experience is still lacking. An interesting perspective in patients with CP is the use of oxytocin, an hypothalamic peptide involved in the reduction of food intake and energy balance, with potential beneficial effects on body composition, and it is currently investigated as an intranasal drug for the treatment of obesity, including patients with PWS (116). Patients with CP and anterior hypothalamic damage may present with abnormal dynamic oxytocin secretion (117), and reduced oxytocin release has been associated with reduced emotion and social cognition (118). A preliminary experience with oxytocin treatment in patients with childhood-onset CP provided encouraging results in terms of neuropsychological and weight characteristics (119).

According to a recent systematic review of the American Academy Sleep Medicine, bariatric surgery may be proposed in obese patients with OSA to reduce important cardiovascular risk factors, like high BMI, DM, and hypertension, with a positive impact on sleep parameters, including AHI, snoring, and sleepiness (120). There is still limited experience with bariatric surgery in patients with CP. In a review of 21 cases who underwent bariatric surgery, maximal mean weight loss was achieved by Roux-en-Y gastric by-pass (RYGB), with an ongoing weight loss at 12 months (mean 33.7 kg), contrasting with a tendency to regain weight at 12 months after sleeve gastrectomy (121). A subsequent review of available studies further supported RYGB as a preferable option in patients with CP based on superior outcomes, which appear to be similar to unselected obese patients (109). Because only a small series is available and long-term follow-up is still lacking, bariatric surgery is currently limited to selected, mostly adult, patients with CP, and no recommendations are available concerning optimal age, timing, and general health conditions for surgery.

#### The Role of Central Stimulants

Central stimulants may simultaneously target HO and SDs. Stimulating drugs include dextroamphetamine, methylphenidate, mazindol, and caffeine/ephedrine, which may ameliorate the consequences of hypothalamic damage on body weight and on sleep-wake cycle through inhibition of the reuptake of dopamine, norepinephrine, and serotonin, and increased release of these monoamines. A recent review evaluated their effects on body weight in few studies/case reports published and reported high percentages of weight reduction or stabilization in patients with CP: 88.2% for dextroamphetamine, and 100% for methylphenidate, mazindol, and caffeine/ephedrine (5). However, few data have been reported on SDs. Dextroamphetamine at low dose (5 mg twice daily) with a median therapy of 13 months was found to improve weight control (stabilization or weight loss) and/or daytime somnolence in 12 patients with childhood-onset CP (22). All the patients affected by daytime somnolence (8/12) improved during treatment, including 2 patients without beneficial effect on weight. Only one patient reported insomnia as adverse event, which was resolved by omission of the evening dose (22).

### Sleep Medicine Strategies

EDS has a multifactorial basis in patients with CP and SBDs, central hypersomnias, and non-adherence with drug therapy (6) may play a critical role in inducing somnolence. In addition, for patients with CP and EDS, clinicians should confirm effective sleep hygiene and acceptable sleep opportunity. The American Academy of Sleep Medicine suggests a psychoeducational approach aiming to highlight habits that may adversely affect sleep and vigilance, and ameliorate approaches (so called “sleep hygiene rules”) to avoid sleep fragmentation and EDS (122).

Few data are available regarding the approach of SBDs in patients with CP. Crowley et al. (6) reported benefit from PAP in 6 out 13 (46%) patients with CP and OSA. We found only a further small case series or single case report on PAP in patients with CP lacking data regarding efficacy and adherence (7, 123).

Stimulants have been used for fatigue and sleepiness in several neurological disorders. Although fatigue and EDS are common problems in patients with CP, there are few data regarding their treatment (6). Modafinil potentiates brain dopaminergic signals *via* dopamine transporter inhibition by acting at the same binding site of cocaine (124). A small case series demonstrated a positive effect of modafinil on EDS in patients with CP and secondary narcolepsy or OSA with residual sleepiness under PAP or not compliant with PAP use (6, 53). Besides modafinil, two other stimulants have been approved for narcolepsy and OSA with residual sleepiness: pitolisant and solriamfetol. Pitolisant is a first-in-class drug acting on histamine H3 receptors (H3Rs) as an antagonist/inverse agonist (125). Very recently, Cordani et al. (123) described a 19-year-old patient with CP and secondary narcolepsy responsive to pitolisant. Lastly, solriamfetol is a new dual dopamine and norepinephrine reuptake inhibitor approved for EDS in narcolepsy and OSA (126), with no clinical data on CP.

Some reports demonstrated fragmented sleep-wake cycles, EDS, and reduced melatonin levels in obese patients with CP (20, 29, 34). In a narrow sample, melatonin intake in 10 adults after CP therapy as children improved EDS and the amount of physical activity (60). On the contrary, other studies failed to demonstrate melatonin efficacy, particularly in patients who meet criteria for narcolepsy or hypersomnia (20). Lastly, Pickering et al. (30) described normal melatonin secretion in 40% of CP survivors, where its supplementation may be not effective.

However, given the probable effects on EDS of sleep habits, PAP, melatonin, and stimulants in comorbid SDs of CP survivors, future studies will be necessary to evaluate their potential role in managing EDS and sleep complaints in patients with CP.

## Conclusion

Sleep disorders represent an important issue in patients with CP. Almost 70% of complaints of SDs and/or EDS are due to SBDs, central hypersomnias, and CRSWDs. SDs may affect QoL and increase respiratory and cardiovascular morbidity and long-term mortality. The main factor involved in SDs is HS due to direct hypothalamic tumor-related damage and/or complications of treatment. Despite the growing data regarding SDs and CP, sleep evaluation is still not routinely proposed, so SDs are often overlooked and undertreated in these patients. Although SDs are strongly related to the presence of HO and neuroendocrine dysfunction, which require dedicated long-life endocrinological management, there is an overwhelming body of evidence that supports the need of sleep management in patients with CP. Adequate sleep quality is crucial throughout the entire lifespan, and patients with CP may impair their QoL because of SDs and EDS. Nevertheless, despite this widely accepted clinical association, large clinical studies to improve clinical practice are lacking, and future studies are urgently needed. We, therefore, suggest to obtain clinical evaluation of sleep habits and SDs in clinical practice from CP survivors and their bed partners, and recommend an adequate diagnostic and therapeutic approach when SDs (SBDs, central hypersomnia, and CRSWDs) are suspected. Finally, considering the positive impact on EDS of sleep hygiene, ventilation, melatonin, and stimulants in comorbid SDs, future studies should be performed to clarify their potential role in managing EDS and sleep alterations in patients with CP. The management of patients suffering from HO and SDs should be multidisciplinary, and the development of new drugs for either condition may hopefully lead to bidirectional positive effects.

## Author Contributions

AR and M-LJ-R conceived, designed the study, revised, and edited the final version of the manuscript. AR, TF, SC, and M-LJ-R wrote the manuscript with contributions by MD, CC, and GV. GP contributed with data and references search, organization, and illustrations. MC and TF contributed with technical assistance to sleep explorations in illustrating cases. DC and VE critically reviewed the manuscript. All authors contributed to the article and approved the submitted version.

## Funding

This work was funded by Neuromed IRCCS, Current Research, by the Italian Ministry of Health.

## Conflict of Interest

The authors declare that the research was conducted in the absence of any commercial or financial relationships that could be construed as a potential conflict of interest.

## Publisher's Note

All claims expressed in this article are solely those of the authors and do not necessarily represent those of their affiliated organizations, or those of the publisher, the editors and the reviewers. Any product that may be evaluated in this article, or claim that may be made by its manufacturer, is not guaranteed or endorsed by the publisher.
